# Supplementary figures and images for: Human Population Density Influences Genetic Diversity of Two Rattus Species Worldwide: A Macrogenetic Approach
Source: Genes (Basel). 2023 Jul 14;14(7):1442. doi: 10.3390/genes14071442 (PMC10379283; doi:10.3390/genes14071442)

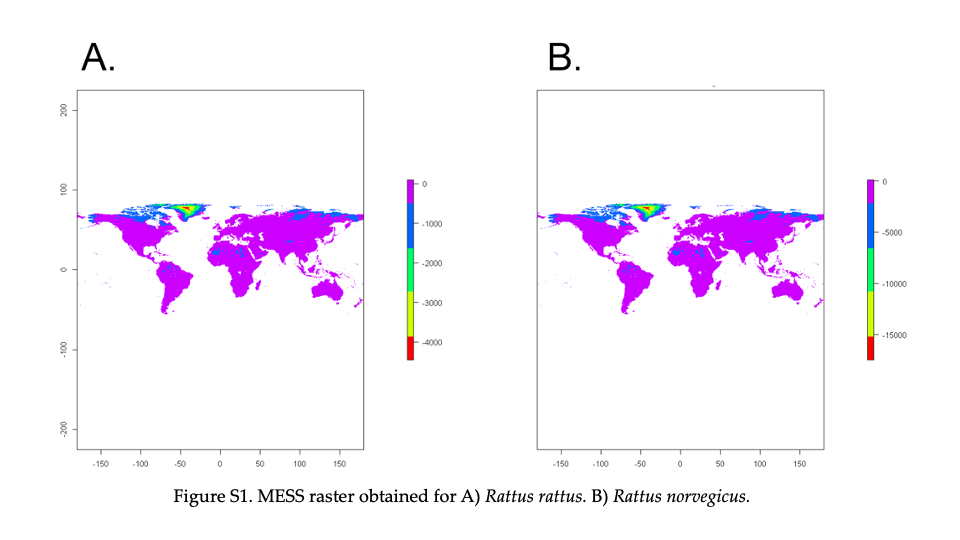

Supplement: Supplementary file 1 [file genes-14-01442-s001.zip › Figure_S1.tiff]

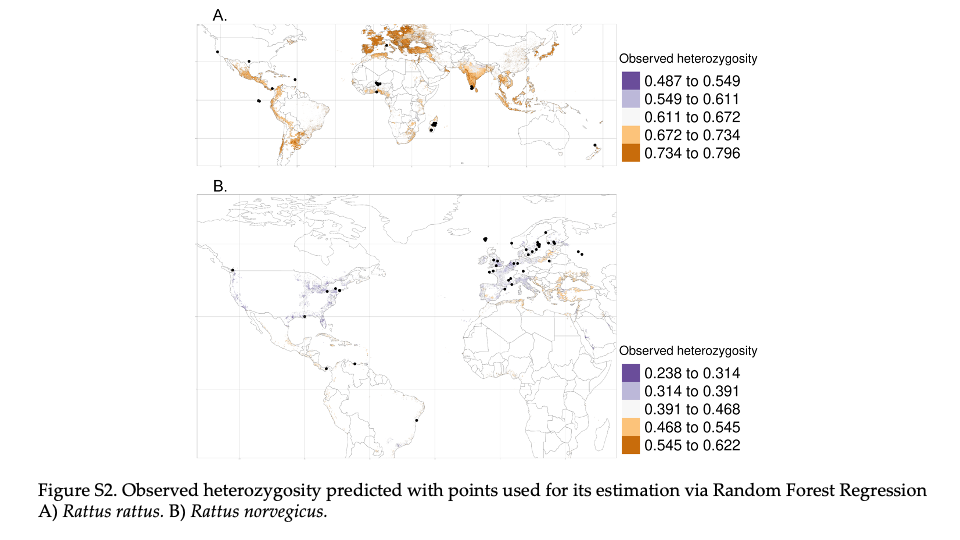

Supplement: Supplementary file 1 [file genes-14-01442-s001.zip › Figure_S2.tiff]
